# The Growing Need to Understand Very Early Onset Inflammatory Bowel Disease

**DOI:** 10.3389/fimmu.2021.675186

**Published:** 2021-05-26

**Authors:** Hengqi B. Zheng, M. Teresa de la Morena, David L. Suskind

**Affiliations:** ^1^ Division of Gastroenterology and Hepatology, Seattle Children’s Hospital, Seattle, WA, United States; ^2^ Department of Pediatrics, University of Washington, Seattle, WA, United States; ^3^ Division of Immunology, Seattle Children’s Hospital, Seattle, WA, United States

**Keywords:** very early onset IBD (VEOIBD), primary immunodeficiencies (PID), genetic testing, IPEX (immune dysregulation), next generation (deep) sequencing (NGS)

## Abstract

Very Early Onset Inflammatory Bowel Disease (VEO-IBD) represents a cohort of inflammatory bowel disease (IBD) patients diagnosed before 6 years of age. Unlike IBD diagnosed at older ages, VEO-IBD can be associated with underlying primary immunodeficiencies. VEO-IBD has been linked to monogenic variations in over 70 genes involved in multiple pathways of immunity. As sequencing technologies and platforms evolve and become readily available, an increasing number of genes linked to VEO-IBD have emerged. Although monogenic defects are rare in VEO-IBD, diagnosis of these variants can often dictate specific treatment. In this mini-review, we set out to describe monogenic variants previously characterized in multiple patients in the literature that contribute to VEO-IBD, diagnostic tools, unique treatment modalities for specific genetic diagnoses, and future directions in the field of VEO-IBD. Although this mini-review is by no means comprehensive of all the novel monogenic variants linked to VEO-IBD, we hope to provide relevant information that is readily accessible to clinicians and educators.

## Introduction

Inflammatory bowel disease (IBD) which includes ulcerative colitis, Crohn disease, and indeterminate IBD, are autoimmune diseases of the gastrointestinal tract. While the etiopathogenesis of IBD is not fully elucidated, IBD is believed to be a result of an exaggerated host inflammatory response to the resident intestinal microbiome. Both genetic and environmental factors have been implicated in the development of IBD (1). IBD diagnosed during childhood or adolescence comprises a fourth of all diagnosed IBD (2). Very Early Onset Inflammatory Bowel Disease (VEO-IBD) represents a subgroup of pediatric IBD diagnosed before the age of 6 years (3) with further subclassification into infantile-onset IBD when diagnosed before 2 years of age and neonatal-onset if diagnosed by 28 days of age (3). Genome-wide association studies (GWAS) reveal the involvement of over 260 loci in IBD. While the associated genotypic mutations are known, the mechanism by which most mutations contribute to disease phenotype development remains to be understood (1, 4). In older children and adults, IBD is more likely polygenic (1). In contrast, VEO-IBD can be rarely associated with monogenic mutations which is often linked to inborn errors of immunity (5). Depending on the patient population, parental consanguinity can be associated with higher risk for genetic mutations in infantile and neonatal forms of IBD. Parental history of consanguinity should prompt a heightened level of suspicion for underlying monogenic variation of VEO-IBD (6, 7). Over 70 genes have been causatively linked to VEO-IBD (3) and additional genetic candidates are being discovered as sequencing technology become faster and provide wider genomic coverage.

The incidence of pediatric IBD is between 2 to 12 cases annually per 100,000 children in industrialized countries (8–10). Over the past decade, the incidence of pediatric IBD and VEO-IBD has continued to rise (8). VEO-IBD represents the fastest growing age group of IBD in some countries. A recent Canadian multi-province population study showed a rising incidence of VEO-IBD (6 months to 5 years) by about 7% per year (10). Thus far, the etiology behind the rise in incidence in pediatric IBD and VEO-IBD is unclear and further research efforts are needed to alleviate disease and healthcare burden.

### Monogenic Variants of VEO-IBD

There is a growing number of causative monogenic variants of VEO-IBD. These monogenic variants are clinically important and dictate divergent pathways in treatment of VEO-IBD. Monogenic etiologies may underlie 15-20% of VEO-IBD patients (3, 11–14). To date, the monogenic variants can be classified into 5 distinct groups: (1) Epithelial barrier defects; (2) Phagocytic defects; (3) T and B cell defects; (4) T regulatory cells and signaling; and (5) Hyper- and auto-inflammatory conditions (see **Table 1**) (3, 11–13).

**Table 1:** Genetic and clinical manifestations in VEO-IBD.

GROUP	GENE DEFECT	CLINICAL PHENOTYPE	SPECIFIC TREATMENT
**Epithelial Barrier Dysfunction**			
NEMO deficiency	*IKBKG*	Ectodermal dysplasia, antibody deficiency, enterocolitis	
ADAM17 deficiency	*ADAM17*	Erythematous psoriasiform rash, dermatitis, enteropathy	
TTC7A deficiency	*TTC7A*	Intestinal atresia, enterocolitis, SCID	
Dystrophic epidermolysis bulls	*COL7A1*	Dystrophic epidermolysis bulbosa, esophageal strictures, Crohn’s disease	
Kindler syndrome	*FERMT1*	Skin blistering, atrophy, cancer, ulcerative colitis	
Loeys-Dietz syndrome	*TGFBR1 and 2*	Skeletal abnormalities, craniofacial abnormalities, vascular injury, enterocolitis	
Congenital chloride diarrhea	*SLC9A3*	Infantile diarrhea, enteropathy with electrolyte abnormalities	
Familial diarrhea	*GUCY2*	Secretory diarrhea, enteropathy with electrolyte abnormalities	
**Phagocytic Defects**			
Chronic Granulomatous Disease (CGD)	*CYBB, CYBA, NCF1, NCF2, NCF4*	Severe bacterial and fungal infections throughout the body, fistulizing Crohn’s Disease with granulomas, intestinal obstruction, perianal abscesses	Anti-microbial prophylaxis, Anti-IL1 blockade, HCT
Leukocyte Adhesion deficiency (LAD1)	*ITGB2*	Delayed separation of umbilical cord, infection, leukocytosis, enteropathy	Anti-microbial prophylaxis, HCT
**T and B Defects**			
Severe Combined Immunodeficiency (SCID)	*ARTEMIS, ZAP70,RAG1/2, IL2RG, ILRA7, LIG4, JACK3, ADA*	Recurrent infections, variable enteropathy and enterocolitis	HCT
Omenn syndrome	*IL7R*	Diffuse erythroderma, lymphadenopathy, eosinophilia, cartilage and hair hypoplasia, hepatomegaly intestinal inflammation	HCT
CTLA-4 deficiency	*CTLA4*	Type 1 diabetes, cytopenias, respiratory infections, enteropathy	Gammaglobulin, Abatacept, Sirolimus
ICOS deficiency	*ICOS*	CVID with recurrent viral and bacterial infections, splenomegaly, colitis	Gammaglobulin
Bruton’s or X-linked agammaglobulinemia	*BTK*	Sinusitis, acute otitis media, colitis	Gammaglobulin
Wiskott-Aldrich syndrome	*WAS*	Thrombocytopenia, eczema, eosinophilia, colitis	HCT
**T regulatory cells and regulatory pathway defects**			
IPEX syndrome	*FoxP3*	Type 1 diabetes, dermatitis, skin and respiratory infections, enteropathy,	Cyclosporin, Tacrolimus, Sirolimus, anti-TNF, HCT
IPEX-like syndrome	*STAT1, STAT3, LRBA, IL2RA, *	STAT1 and STAT3: enteropathy, severe viral and bacterial infections, and endocrinopathy.	JAK inhibitors, Sirolimus, HCT
		LRBA: enteropathy, cytopenias, lymphadenopathy, and hepatosplenomegaly.	
		IL2RA: enteropathy, eczema, recurrent viral infections, and autoimmune anemia	
IL-10 signaling defects	*IL-10RA, IL-10RB*	Colitis, folliculitis, perianal fistulas, B cell lymphoma, occasional arthritis	HCT
**Hyperinflammatory and auto inflammatory defects**			
X-linked lymphoproliferative syndrome 2	*XIAP*	Splenomegaly, hemophagocytic lymphohistiocytosis (HLH), fistulizing enteropathy	HCT
NLRC4	*NLRC4*	Infantile enterocolitis, autoinflammation	Anti-IL18 blockade
Mevalonate kinase deficiency	*MVK*	Episodic fevers, peritonitis, arthritis, bloody enterocolitis	Anti-IL1 blockade
NOD2 signaling defect	*TRIM22*	Perianal fistulizing disease, enterocolitis

1. Epithelial barrier defects can result in inflammation of the skin and intestines early on in life. The *IKBKG* gene encodes the regulatory protein NEMO on the X chromosome which is a subunit of the of NF-kB kinase (IKK) complex needed for activation of the NF-kB family of transcription factors and X-linked ectodermal dysplasia and antibody deficiencies occurs in the absence of a functional NEMO protein (15). *ADAM17* loss-of-function mutation leads to a lack of disintegrin and metalloproteinase 17 which converts TNF-alpha from membrane-bound form to soluble TNF-alpha (16). Clinical manifestations of ADAM17 deficiency can lead to erythematous psoriasiform rash, dermatitis, and diarrhea (16). *TTC7A* gene encodes for a regulatory protein which with EFR3 homolog B to regulate phosphatidylinositol kinase to maintain plasma membrane homeostasis (17). TTC7A deficiency can present in the neonatal period with intestinal atresia, apoptotic enterocolitis, and severe combined immunodeficiency (SCID) (17). Dystrophic epidermolysis bullosa and Crohn’s disease can occur with mutations in the *COL7A1* which encodes for collagen VII (18). Kindler syndrome results from mutations in the *FERMT1* gene whose gene product are adhesion proteins binding cytoplasmic domains and clinically presents with skin blistering, atrophy and cancer along with ulcerative colitis (19–21). Loeys-Dietz Syndrome results from transforming growth factor beta receptors (TGFBR) 1 and 2 mutations leading to cytokine signaling defects manifesting as severe IBD, skeletal abnormalities, craniofacial abnormalities, and vascular injury (22). Aberrations in the *SLC9A3* gene which encodes for intestinal chloride/bicarbonate exchanger, leads to congenital chloride diarrhea, and gain of function *GUCY2* which is an intestinal receptor for bacterial enterotoxins, leads to familial diarrhea disorders (23, 24).
2. Phagocytic defects can lead to alterations in pathogen recognition and clearance. Chronic granulomatous disease (CGD) is a clinical manifestation of defective phagocytic activity in pathogen killing and clearance. Components of the nicotinamide adenine dinucleotide phosphate (NADPH) oxidase complex [gp91-phox (*CYBB*), p22-phox (*CYBA*), p47-phox (*NCF1*), p67-phox (*NCF2*), p40-phox (*NCF4*)] are defective, most commonly in granulocytes (25). Clinical manifestations of CGD include wide phenotypic variations with severe bacterial and fungal infections throughout the body, cutaneous and deep organ abscesses, and intestinal inflammation appearing similar to fistulizing Crohn’s disease with microscopic granulomatous colitis (26). Intestinal obstruction and strictures along with perianal abscesses and fistulae can be present as non-infectious manifestations of CGD (27, 28). Leukocyte adhesion defect 1 (LAD1) results from a mutation in *ITGB2* gene encoding CD18, a subunit of the beta2 integrin leading to impairment of leukocyte migration (29). LAD1 is associated with an IBD phenotype, severe infection, delayed umbilical cord separation, and a high reactive leukocytosis consistent with immobile peripheral granulocytes (29).
3. T and B cell defects within the adaptive immune system can present with intestinal inflammation. Mutations in genes that affect the development or function of T and B cells [*DCLRE1C(ARTEMIS), ZAP70, RAG1/2, IL2RG, ILRA7, LIG4, JAK3, ADA*] can result in intestinal inflammation along with recurrent infections from severe combined immunodeficiency (SCID) (30–32). Omenn syndrome is an autosomal recessive type of SCID with residual T cell function manifesting severe eczema, hair and cartilage hypoplasia, and intestinal inflammation with defects in IL7R (33, 34). Cytotoxic T-lymphocyte antigen 4 (*CTLA4*) encodes for a protein receptor which functions as a negative regulator of T regulatory cells (35). CTLA4 protein deficiency leads to a lack of self-tolerance and hyperinflammatory pathway manifesting in enteropathy, cytopenia, type 1 diabetes, and respiratory symptoms (35). Mutations in the *ICOS* gene encoding for costimulatory receptor for inducible T cell activation can present with common variable immunodeficiency (CVID) resulting in recurrent bacterial and viral infections, splenomegaly, and colitis (36, 37). Bruton’s or X-linked agammaglobulinemia with a defect in the Bruton’s tyrosine kinase (*Btk*) gene leads to sinusitis, acute otitis media, and colitis (38). Wiskott-Aldrich Syndrome (WAS) occurs with a mutated WAS gene leading to a reduced or absent WAS protein expression which leads to actin filament defect in hematopoietic cells (39). WAS patients can present with the classic findings of thrombocytopenia, eczema, eosinophilia, and immune deficiencies along with colitis (39).
4. T regulatory (Treg) cells and the regulatory pathway play a critical role in the maintenance of intestinal homeostasis. Forkhead box protein 3 (FOXP3) is a critical transcription factor for CD4+ Foxp3+ Tregs and pathogenic variants in FOXP3 leads to immunodysregulation, polyendocrinopathy, enteropathy X-linked (IPEX) syndrome and prototype of a group of disorders called PIRD (Primary Immune Regulatory Disorders) (40, 41). Over 63 *FOXP3* mutations have been reported though severity of disease is independent of level of protein expression. Classic IPEX syndrome presents as the clinical triad of profuse diarrhea (autoimmune enteropathy), type-1 diabetes (endocrinopathy), and dermatitis with a propensity for skin and respiratory infections (42). In addition to classical presentation, variable clinical manifestations of IPEX patients show no apparent genotype-phenotype correlation and disease course or outcome (43). Other inborn errors of immunity can manifest in an IPEX-like manner with normal FOXP3 expression, associated immune dysregulation, eczema, and enteropathy (44). Gain of function mutations in signal transducer and activator of transcription1 (*STAT1*) and 3 (*STAT3*) can lead to enteropathy, severe viral and bacterial infections, and endocrinopathy (44). LPS-responsive beige-like anchor (*LRBA*) mutation leads absent or dysfunctional protein needed for Treg function and can present with enteropathy, cytopenias, lymphadenopathy, and hepatosplenomegaly (44). *IL2RA* mutations leads to CD25 protein deficiency and impaired Treg function, resulting in enteropathy, eczema, recurrent viral infections, and autoimmune anemia (45). Defects in the IL-10 pathway were some of the first monogenic variants discovered to be associated with VEO-IBD especially with presentation during the neonatal period (46, 47). IL-10 is a regulatory cytokine which limits secretion of proinflammatory cytokines and acts on multiple innate immune cells such as macrophages, monocytes, and Natural Killer (NK) cells along with adaptive immune cells (48). Aberrations in the IL-10 signaling pathway leaves pro-inflammatory pathways unopposed and can manifest as severe colitis, perianal fistulas, folliculitis, and occasional arthritis (46–48). The IL-10 hetrotetrameric receptor consists of two alpha subunits (IL-10R1) and two beta subunits (IL-10R2) and results in downstream activation of signal transducer and activator of transcription (STAT-3) (48). Mutations in the genes *IL-10RA* and *IL-10RB* which encode for IL-10R1 and IL-10R2 are defects leading to infant onset-IBD (46, 47). IL-10R deficiency patients have been reported to develop large B-cell lymphoma through a mechanism that is yet to be fully elucidated but thought to be due to an impaired immune surveillance (49, 50).
5. Autoimmune and hyperinflammatory systemic conditions can manifest with intestinal inflammation. X-linked inhibitor of apoptosis (XIAP) deficiency or X-linked lymphoproliferative syndrome type 2 (XLP-2) can result from various mutations within domains of *BIR2* (51, 52). XIAP interacts with the serine/threonine-protein kinase 2 (RIPK2) which further mediates nuclear factor kB (NF-kB) for immune cell activation, inhibits caspase activity, and functions in the nucleotide-binding oligomerization domain-containing 1/2 (NOD1/2) intracellular pattern recognition receptor signaling pathway for maintaining intestinal homeostasis with the microbiota through the innate immune system (53, 54). Clinically, XIAP patients have recurrent splenomegaly, hemophagocytic lymphohistiocytosis (HLH) triggered by Epstein-Barr virus (EBV), and 30% with fistulizing IBD (54, 55). Mutations in *NLRC4* have been reported in patients with infantile enterocolitis and autoinflammation due to constitutive inflammasome activation and production of both IL1B and IL18 (56, 57). Mevalonate kinase deficiency results with mutations in the *MVK* gene which leads to an accumulation of substrate mevalonate (58). Clinical symptoms manifest with a pro-inflammatory cytokine response mediated by IL-1b with episodic fevers, peritonitis, arthritis, and bloody enterocolitis (58). Tripartite motif-containing 22 (*TRIM22*) encodes for ubiquitin ligase which plays a role in differentiation of lymphocytes and found in macrophages and interacts with the NOD2 pathway (59). Through whole exome sequencing methods, *TRIM22* mutations were recently recognized in as a causative variant in VEO-IBD with perianal disease and fistulas (59).

## Evaluation

VEO-IBD with underlying genetic mutation can present a diagnostic challenge. Significant time can lapse from symptom onset to final diagnosis. A high index of suspicion is required for patients presenting early in life. An expedited evaluation is required for these patients including comprehensive exam, laboratory, endoscopic, histologic, and genetic workups. A multi-disciplinary approach is required for a comprehensive evaluation of these patients including input from gastroenterology, immunology, genetics, and nutrition. A thorough evaluation is crucial especially in neonatal- and infantile-onset IBD as monogenic etiologies underlie up to 20% of VEO-IBD patients (12, 13, 60). Basic laboratory studies including complete blood count (CBC) with differential, inflammatory markers (CRP, ESR), comprehensive metabolic panel, and stool studies are often the first line of workup. Immunological panels to evaluate T cell population and memory subsets, B cell subsets, and NK cells by flow cytometry along with immunoglobulin levels and response to vaccinations can detect classic defects in adaptive immunity. A neutrophil oxidative burst assay using dihydrorhodamine identifies patients with chronic granulomatous disease. Flow cytometric based assays can also be done with intracellular protein expression looking for deficiencies in FOXP3 leading to IPEX and XIAP especially in male patients (61, 62). If available, cytokine and chemokine panels (IL-1, IL-2, IL-6, IL-10, IL-18, TNF-alpha, IFN-gamma) can be used to identify specific pathway defects, target treatment, and detect complications such as HLH development in XIAP (63, 64).

Endoscopy with histologic evaluation *via* hematoxylin and eosin (H+E) stain aids in the diagnosis of VEO-IBD though lacks diagnostic specificity. The likelihood of an underlying genetic variant increases if enteritis with small bowel involvement or colitis with perianal disease is present within the VEO-IBD cohort as compared to the VEO-IBD patients with isolated colitis (65). Currently there are limited pathognomonic histological findings to suggest a monogenic cause in VEO-IBD though studies describe the increased presence of apoptosis, severe chronic architectural changes, villous blunting, and abundance of eosinophils with VEO-IBD as compared to older onset pediatric IBD (66). Some histopathological findings for specific primary immunodeficiencies should be considered when reviewing biopsies. SCID microscopic features include the presence of epithelial cell apoptosis, eosinophilic and neutrophilic infiltrations with villous atrophy (16). CGD histology may contain multiple non-caseating granulomas with minimal surrounding inflammation (13). IPEX enteropathy can endoscopically and histologically appear as villous blunting and chronic inflammation within the lamina propria (42).

## Treatment

Given the brevity of this mini-review, we aim to describe therapies for a few monogenic defects in VEO-IBD. In general, therapeutic options are patient specific and are influenced by underlying genetic variation or primary immunodeficiency. Interventions can include nutritional therapies, immunosuppression, as well as hematopoietic stem cell transplantation depending on the underlying genetic variations or primary immunodeficiencies (3, 11, 12, 67). In addition to nonspecific immunosuppression such as corticosteroids, some targeted treatment options exist and depend on timely diagnosis of the underlying immunologic dysfunction. Therapy for IPEX has included a range of immunosuppression regimens including yclosporin, tacrolimus, anti-TNF, and mammalian target of rapamycin (mTOR) inhibitors. Risk of infection is balanced against level of immunosuppression required to control systemic inflammation (42). Hematopoietic cell transplantation (HCT) has been performed in IPEX patients (42). In addition, anti-IL1 blockade has also been used in CGD patients with high levels of IL-1 with variable success (68, 69). Anti-microbial prophylaxis including anti-fungal and anti-bacterial medications is needed in patients with CGD and LAD1 (27, 28). HCT is the definitive therapy for phagocytic defects (70). HCT is also a treatment modality for treatment refractory XIAP and deters the risk of developing of hemophagocytic lymphohistiocytosis (HLH) (51). Mevalonate kinase deficiency (Hyper IgD syndrome) may also benefit from anti-IL1 blockade in efforts to temper the pro-inflammatory cascade (59). The diagnosis of SCID, Omenn syndrome, and WAS requires early intervention with HCT prior to the emergence of complications (39). The gastrointestinal manifestations of CTLA-4 haploinsufficiency and LRBA deficiency can be treated with gammaglobulin infusions and a number of immunomodulating agents targeting the activation of T-cells. Sirolimus, a mammalian target of rapamycin has been used successfully with caution though potential side effects profile of CMV reactivation, respiratory infections and sepsis are reported in CTLA-4 (35). Abatacept, a fusion protein containing the extracellular domain of CTLA-4, has been used successfully in patients with CTLA4 haploinsufficiency and as long-term therapy for those with LRBA deficiency (71). In severe cases, CTLA 4 may require HCT (35).

## Advancement in Diagnostic Testing

Making the correct genetic diagnosis is imperative as therapeutic interventions depends on targeting the underlying immune dysregulation. Timely identification of specific monogenic variants of VEO-IBD represents a diagnostic paradigm for patient specific precision medicine. To date, multiple sequencing techniques are offered to identify monogenic IBD and are more accessible with the advent of next-generation sequencing (NGS).

Targeted gene panels (TGP) have been developed and employed at institutions nationally. Depending on the selection and number of genes included, panels sequencing can offer high coverage of the most likely genetic culprits with a high diagnostic accuracy (72). Whole exome sequencing (WES) provides higher coverage sequencing of coding regions of the genome at higher costs depending on depth of reads. There may be some limitations in utilizing commercially available WES as some genes such as *IKBKG* associated with NEMO deficiency and *NCF1* associated with CGD may have pseudo gene loci (60, 73, 74) Whole genome sequencing (WGS) offers the ability to capture further sequencing with the capacity to pick up copy number variations and examine noncoding areas with promoters and enhancers (72, 75, 76). However, without the availability of large research centers, WES and WGS can be difficult to come by and costly to perform.

The advent of RNA-sequencing offers a glimpse into how transcriptomics may transform our diagnostic capacity. RNA sequencing can offer target organ tissue level gene expression as certain cell types (epithelial cells, Paneth cells, M cells etc.) exist predominantly within the gastrointestinal tract. With drop-seq (77), single-cell RNA-sequencing (scRNA-seq) brings the capability to interrogate individual cellular transcripts to determine pathogenic pathways even within rare cellular subpopulations previously inaccessible using conventional techniques. A recent scRNA-seq study describes a cellular module called GIMATS (consisting of IgG plasma cells, phagocytes, activated T cells, and stromal cells) could indicate resistance to anti-TNF therapy in adult IBD patients (78). Currently both bulk and single-cell RNA-sequencing are still in the research stages of discovery but have the potential to reveal further genetic defects.

## Conclusion

VEO-IBD represents a unique group of pediatric IBD patients diagnosed in children less than 6 years of age. These patients require a focused and multidisciplinary approach that can harmonize expertise between gastroenterology, immunology, and genetics (79). Rapid innovations in genetic molecular sequencing techniques have identified novel mutations and genetic linkage of phenotype and clinical manifestations. VEO-IBD highlights the importance and need for precision medicine in the clinical setting of IBD care.

## Author Contributions

HZ wrote the first draft of the manuscript. DS and MM wrote sections of the manuscript. All authors contributed to the article and approved the submitted version.

## Conflict of Interest

The authors declare that the research was conducted in the absence of any commercial or financial relationships that could be construed as a potential conflict of interest.
